# Prediction and clinical impact of delayed lymphopenia after chemoradiotherapy in locally advanced non-small cell lung cancer

**DOI:** 10.3389/fonc.2022.891221

**Published:** 2022-08-18

**Authors:** Byung-Hee Kang, Xue Li, Jaeman Son, Changhoon Song, Hyun-Cheol Kang, Hak Jae Kim, Hong-Gyun Wu, Joo Ho Lee

**Affiliations:** ^1^ Department of Radiation Oncology, Seoul National University College of Medicine, Seoul National University Bundang Hospital, Seongnam, South Korea; ^2^ Department of Radiation Oncology, Seoul National University College of Medicine, Seoul National University Hospital, Seoul, South Korea

**Keywords:** non-small cell lung cancer, chemoradiotherapy, lymphopenia, clinical predictor, dose-volume histograms, prediction nomogram

## Abstract

**Introduction:**

The dosimetric factors of radiotherapy have an acute impact on the host immune system during chemoradiotherapy (CRT) in locally advanced non-small cell lung cancer (NSCLC). However, even after CRT, a substantial number of patients remain immunosuppressed with delayed lymphopenia. Therefore, we aimed to evaluate clinical and dose-volumetric predictors of delayed lymphopenia after CRT in locally advanced NSCLC.

**Materials and methods:**

We retrospectively reviewed 272 patients with locally advanced NSCLC who received definitive CRT from January 2012 to August 2020. Differential blood count data, including serum albumin values, were obtained at baseline, during and at first follow up after CRT. Acute and delayed lymphopenia events were defined as grade III/IV lymphopenia developed during or 4-12 weeks after CRT completion, which accounted for 84% and 10% of cases, respectively. Dose-volume histogram parameters for planned target volume, whole body, heart, lung, great vessels, spleen, esophagus and thoracic vertebral bodies were evaluated.

**Results:**

Multivariate analysis revealed that patients with delayed lymphopenia were associated with inferior overall survival (HR 2.53, *P* = 0.001) and progression-free survival (HR 1.98, *P* = 0.006). However, there was no significant survival difference between groups stratified by acute lymphopenia. On multivariable logistic regression models, lung V5, baseline ALC, during-CRT ALC, and albumin nadir were significant predictors for delayed lymphopenia. Furthermore, the nomogram for delayed lymphopenia based on these variables had good discrimination (area under the curve, 0.905).

**Conclusions:**

In this study, we investigated the prognostic significance of delayed lymphopenia and identified clinico-dosimetric parameters to predict delayed lymphopenia.

## Introduction

Non-small cell lung cancer (NSCLC) continues to be a leading cause of cancer death worldwide, and approximately 30% of patients are first diagnosed with stage III locally advanced disease (1). Recently, consolidation immunotherapy has shown survival gain in addition to definitive chemoradiotherapy (CRT) for locally advanced NSCLC (2, 3). Recent advances in immunotherapy have revealed the importance of the immune system in cancer control. As the importance of assessment of immune status in solid cancer patients has become recognized, numerous immune-based biomarkers have been identified (4). Absolute lymphocyte count (ALC) is a solid and clinically relevant biomarker of radiotherapy (RT)-induced immunotoxicity and correlates with survival after RT in multiple solid tumors (4, 5). Early clinical experiences also suggest that ALC is a predictor of response to checkpoint blockade (6–8).

The therapeutic effect of immunotherapy is affected by immune microenvironments, and radiation-induced lymphopenia (RIL) is an unignorable issue for locally advanced NSCLC patients who are candidates for consolidation immunotherapy. Circulating lymphocytes are extremely sensitive to RT. Thus, RIL occurs in 40% to 90% of patients who undergo conventional external beam radiation therapy (5, 9). It is important to evaluate the risk for a severe clinical course of RIL and to develop strategies to mitigate RIL. While previous studies addressing RIL used inconsistent definitions of lymphopenia, most of them defined only acute lymphopenia during and early after treatment (10–13). However, the status of acute lymphopenia can change rapidly after RT (9). Therefore, it is not possible to postulate the survival impact of delayed lymphopenia, especially at the time of consolidation immunotherapy, from the above study findings. Meanwhile, numerous studies have identified clinical and dosimetric predictors associated with RIL in NSCLC (10–12). Nevertheless, there is no clinical consensus for immune organ-at-risk or recommended dose constraints.

Our main hypothesis in the current study is that delayed lymphopenia after CRT will have a detrimental effect on prognosis in locally advanced NSCLC patients receiving definitive CRT. Furthermore, we aimed to identify the clinical and dosimetric variables that act as predictors of delayed lymphopenia.

## Materials and methods

### Patients

The Institutional Review Board of Seoul National University Hospital approved this study (IRB no H-2003-145-1111). This study retrospectively reviewed 272 patients with locally advanced non-small cell lung cancer who underwent definitive CRT from January 2012 to August 2020. Data were collected for patients who had differential blood counts performed at baseline, during CRT, and at 4 to 12 weeks after CRT completion. Patients were included in the study if they had no distant metastases at presentation and had received more than 60 Gy of RT, indicating definitive therapy. All patients were treated with concurrent radiation and chemotherapy (three-dimensional, n = 124; volumetric modulated arc therapy (VMAT), n = 148). Most patients were treated with standard fractionation regimes of either 60 Gy in 30 fractions or 66 Gy in 33 fractions. The other patients (n = 13) were treated with hypofractionated RT; the dose per fraction ranged from 2.25 to 4 Gy, and the delivered dose ranged from 60 to 80 Gy. The most common chemotherapy regimens were weekly carboplatin and paclitaxel (n = 193) and weekly cisplatin and docetaxel (n= 71). No patients received neoadjuvant chemotherapy, and only 30 patients received consolidation immunotherapy.

### Outcome assessment

Differential blood count data, including serum albumin values, were obtained at baseline, during CRT, 0 to 4 weeks, and 4 to 12 weeks after CRT completion. ALC nadir was recorded at each time point and assessed using the Common Terminology Criteria for Adverse Events, version 4.0. After CRT, ALC values were excluded if the patients had received any other anticancer treatments before the ALC count was recorded, excluding immunotherapy. Dose-volume histogram (DVH) parameters for planned target volume, whole body, heart, lung, great vessels, spleen, esophagus and thoracic vertebral bodies were evaluated. DVH parameters for each structure were mean dose (Dmean), maximum dose (Dmax) and the percentage volumes receiving 5 Gy (V5), 10 Gy (V10), 20 Gy (V20), 30 Gy (V30), 40 Gy (V40), 50 Gy (V50), and 60 Gy (V60).

### Statistical analysis

To assess clinical outcomes, Kaplan-Meier curves with log-rank tests and multivariate stepwise Cox regression analyses were constructed for overall survival (OS) and progression-free survival (PFS) rates. Univariate analyses using t-test, Kruskal-Wallis Rank Sum test, and the chi-square test were used to identify clinical variables associated with the delayed lymphopenia. To adjust for multiple comparisons, we applied the false discovery rate (FDR) approach. Univariate and multivariable logistic regression analysis were conducted to identify factors associated with delayed lymphopenia. Predictors with an adjusted P value <0.05 on univariate analysis were included in the multivariate model, and backward elimination was used to obtain the final model. The final model was presented as a nomogram. Model performance was evaluated through discrimination and calibration. The area under the receiver operating characteristic (ROC) curve (AUC) was used to assess the discriminative power of the nomogram. Internal validation with split-sample (70/30), and 100 iterations of tenfold cross-validation, 400 bootstrap resamples were used. The statistical analyses were performed using R software (version 3.5.3; R Foundation for Statistical Computing, Vienna, Austria).

## Results

**Figure 1:**
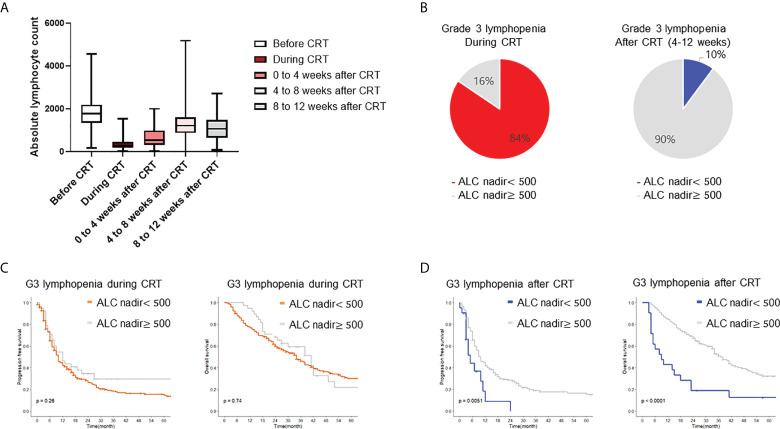
Incidence and survival impact of radiation induced lymphopenia. **(A)** Proportion of lymphopenia during peri-treatment period. Boxplot shows distribution of ALC before, during, after CRT. **(B)** Incidence for grade III/IV lymphopenia during and 4 to 12 weeks after CRT completion. **(C, D)** Kaplan-Meier plot of progression free survival and overall survival. CRT, chemoradiotherapy; ALC, absolute lymphocyte count.

The baseline characteristics for 272 patients are summarized in **Table 1**. Median follow-up was 24 months (range 1–100 months), during which 151 deaths (55%) were reported. **Figure 1A** shows serial rates of ALC throughout CRT for the entire patient cohort. ALC was significantly depleted during CRT and gradually recovered for 2 months after treatment. Severe lymphopenia (ALC nadir < 500 cells/mm3) was found in 84%, 48%, and 10% of cases during CRT, from 0 to 4 weeks, and from 4 to 12 weeks after CRT, respectively (**Figure 1B**). Acute and delayed lymphopenia were defined as ALC < 500 cells/mm3 during and at 4-12 weeks after CRT completion, respectively.

**Table 1:** Patient characteristics and univariate logistic regression analysis for delayed lymphopenia.

Characteristic	Delayed lymphopenia (ALC nadir <500, 4 to 12 weeks after CRT completion)			
	No (N=244)	Yes (N=28)	OR (95% CI)	*P* value ∫
Age, y *	64.0 [59.0;71.0]	71.5 [67.5;76.0]	1.073 (1.025-1.129)	0.004
Sex				
Female	46 (18.9%)	4 (14.3%)	1.000	
Male	198 (81.1%)	24 (85.7%)	1.394 (0.508 -4.913)	0.636
Performance				
ECOG0	5 (2.0%)	0 (0.0%)	1.000	
ECOG1	235 (96.3%)	27 (96.4%)	NA	0.989
ECOG2	4 (1.6%)	1 (3.6%)	NA	0.989
Body weight, kg ∮	64.9 ± 9.9	63.7 ± 9.7	0.988 (0.948-1.028)	0.636
Height, cm *	166.1 [160.9;170.2]	165.8 [162.8;170.0]	1.004 (0.978-1.054)	0.863
Smoking				
Current smoker	101 (41.4%)	7 (25.0%)	1.000	
Ex smoker	90 (36.9%)	15 (53.6%)	2.405 (0.968-6.542)	0.149
Never smoker	53 (21.7%)	6 (21.4%)	1.633 (0.503-5.159)	0.532
Pack year *	30.0 [10.0;40.0]	40.0 [17.5;50.0]	1.017 (1.001-1.034)	0.039
Clinical stage †				
2a/b	7 (2.9%)	2 (7.1%)	1.000	
3a	141 (57.8%)	13 (46.4%)	0.322 (0.069-2.313)	0.352
3b	73 (29.9%)	8 (28.6%)	0.383 (0.075-2.862)	0.500
3c	23 (9.4%)	5 (17.9%)	0.761 (0.129-6.119)	0.834
Histology				
Adenocarcinoma	109 (44.7%)	10 (35.7%)	1.000	
Squamous cell carcinoma	111 (45.5%)	14 (50.0%)	1.817 (0.467-5.952)	0.600
Others	24 (9.8%)	4 (14.3%)	1.375 (0.590-3.315)	0.500
Radiotherapy technique				
3D-CRT	118 (48.4%)	6 (21.4%)	1.000	
VMAT	126 (51.6%)	22 (78.6%)	3.434 (1.425-9.590)	0.010
Chemotherapy				
Docetaxel/Cisplatin	49 (25.9%)	22 (26.5%)	1.000	
Paclitaxel/Carboplatin	133 (70.4%)	60 (72.3%)	10.9 (2.247-196.42)	0.020
Others	7 (3.7%)	1 (1.2%)	10.0 (0.369-272.59)	0.234
Consolidation immunotherapy				
No	215 (88.1%)	27 (96.4%)	1.000	
Yes	29 (11.9%)	1 (3.6%)	0.374 (0.020-1.895)	0.500
PD-L1 expression				
Negative (<1%)	7 (2.9%)	2 (7.1%)	1.000	
Low (1-49%)	141 (57.8%)	13 (46.4%)	1.348 (0.418-4.468)	0.614
High (50-100%)	73 (29.9%)	8 (28.6%)	1.155 (0.277-4.373)	0.833
Not available	23 (9.4%)	5 (17.9%)	0.815 (0.293-2.474)	0.701
Baseline ALB, g/dL *	4.0 [3.8; 4.3]	3.8 [3.2; 4.0]	0.121 (0.051-0.268)	<0.001
Baseline ALC, cells/uL *	1830.3 [1396.8;2271.3]	1295.6 [934.4;1632.3]	0.992 (0.988-0.995)	<0.001
Baseline Hb, g/dL *	13.1 [12.1;14.1]	11.9 [9.7;12.8]	0.569 (0.439-0.726)	<0.001
Baseline PLT, 10^3^/uL *	257.0 [211.5;313.5]	198.5 [188.5;283.0]	0.996 (0.989-1.003)	0.500
Baseline WBC, 10^3^/uL *	7.2 [6.1; 8.9]	6.4 [5.4; 8.5]	0.854 (0.603-1.160)	0.500
Baseline ANC, cells/uL *	4253.0 [3296.0;5620.5]	4259.5 [3411.5;6620.5]	1.000 (0.999-1.000)	0.659
ALB nadir, g/dL *	3.7 [3.4; 3.9]	3.2 [2.6; 3.6]	0.227 (0.100-0.489)	0.001
ALC nadir, cells/uL *	340.8 [226.5;468.8]	194.0 [134.6;268.9]	0.998 (0.997-0.999)	<0.001
Hb nadir, g/dL *	11.3 [10.2;12.2]	9.4 [8.1;11.3]	0.650 (0.520-0.805)	0.001
PLT nadir, 10^3^/uL *	151.0 [125.0;185.0]	134.0 [106.5;183.0]	0.994 (0.989-0.999)	0.095
WBC nadir, 10^3^/uL *	2.8 [2.1; 3.7]	2.3 [1.5; 3.3]	0.922 (0.769-1.082)	0.500
ANC nadir, cells/uL *	1987.5 [1377.0;2678.5]	1530.0 [983.5;2695.0]	1.000 (0.999-1.000)	0.523

OR, odds radio; NA, not available; 3D-CRT, 3-dimensional conformal radiation therapy; VMA, volumetric modulated arc therapy; ALB, albumin; ALC, absolute lymphocyte count; Hb, hemoglobin; PLT, platelet; WBC, white blood cell; ANC, absolute neutrophil; CRT, chemoradiotherapy. † Clinical stage was determined according to American Joint Committee on Cancer staging system, 8th edition * Expressed as median [interquartile range]; Kruskal-Wallis Rank Sum test were used for comparison ∮ Expressed as mean ± SD; Student t-test were used for comparison ∫ FDR adjusted P value.

To assess the survival impact of RIL, patients were stratified by acute and delayed lymphopenia. Kaplan-Meier curves and log-rank tests were analyzed for PFS and OS. Patients with delayed lymphopenia had worse OS (log-rank hazard ratio (HR) = 3.12, p <0.001) and PFS (log-rank HR= 2.14, p <0.001, **Figure 1D**). However, there was no significant survival difference between groups stratified by acute lymphopenia (**Figure 1C**). Multivariable Cox analyses revealed that age, planning target volume (PTV), hemoglobin nadir during CRT, and delayed lymphopenia were significantly associated with PFS. Male sex, larger PTV, lower hemoglobin level during CRT, and delayed lymphopenia were significantly correlated with worse OS (**Table 2**).

**Table 2:** Univariate and multivariate Cox regression analysis for PFS and OS.

	PFS						OS				
	Univariate analysis			Multivariate analysis			Univariate analysis			Multivariate analysis	
	HR (95% CI)	*P* Value		HR (95% CI)	*P* Value		HR (95% CI)	*P* Value		HR (95% CI)	*P* Value
Age, year	0.980 (0.966-0.995)	0.009		0.978 (0.963-0.993)	0.004		1.019 (1.305-1.038)	0.041			
Smoking, pack year	0.996 (0.989-1.002)	0.190					1.010 (1.001-1.017)	0.012			
Sex, male	1.007 (0.714-1.42)	0.970					2.113 (1.002-3.422)	0.002		2.116 (1.286-3.422)	0.007
PTV, cc	1.001 (1.000-1.002)	<0.001		1.001 (1.000-1.001)	0.009		1.002 (0.983-1.002)	<0.001		1.001 (1.001-1.002)	<0.001
EQD2, ≥66Gy	0.776 (0.584-1.030)	0.079					0.648 (0.990-0.905)	0.011			
Clinical T3/T4	1.025 (0.772-1.361)	0.866					1.547 (1.000-2.139)	0.008			
Immunotherapy, yes	0.706 (0.393-1.268)	0.243					0.892 (0.33-1.925)	0.770			
Delayed lymphopenia, yes	2.235 (1.386-3.604)	0.001		1.984 (1.219-3.227)	0.006		3.196 (0.998-5.034)	<000.1		2.531 (1.547-4.140)	0.001
ALB nadir *, g/dL	0.806 (0.591-1.099)	0.173					0.457 (1.119-0.634)	<000.1			
ALC nadir *, cells/uL	0.999 (0.998-1.000)	0.016					0.999 (1.026-1.000)	0.197			
Hb nadir *, g/dL	0.869 (0.791-0.956)	0.004		0.883 (0.802-0.972)	0.011		0.787 (1.197-0.870)	<000.1		0.856 (0.773-0.949)	0.003
PLT nadir *, 10^3^/uL	0.998 (0.995-1.000)	0.072					0.998 (1.401-1.001)	0.131			
WBC nadir *, 10^3^/uL	0.935 (0.838-1.043)	0.227					1.004 (0.764-1.141)	0.947			
ANC nadir *, cells/uL	1.000 (1.000-1.000)	0.245					1.000 (0.873-1.000)	0.765			

PFS, progression-free survival; OS, overall survival; HR, hazard ratio; CI, confidence interval; PTV, planning target volume; EQD2, equivalent dose at 2 Gy per fraction; ALB, albumin; ALC, absolute lymphocyte count; Hb, hemoglobin; PLT, platelet; WBC, white blood cell; ANC, absolute neutrophil; CRT, chemoradiotherapy; * nadir during chemoradiotherapy.

**Figure 2:**
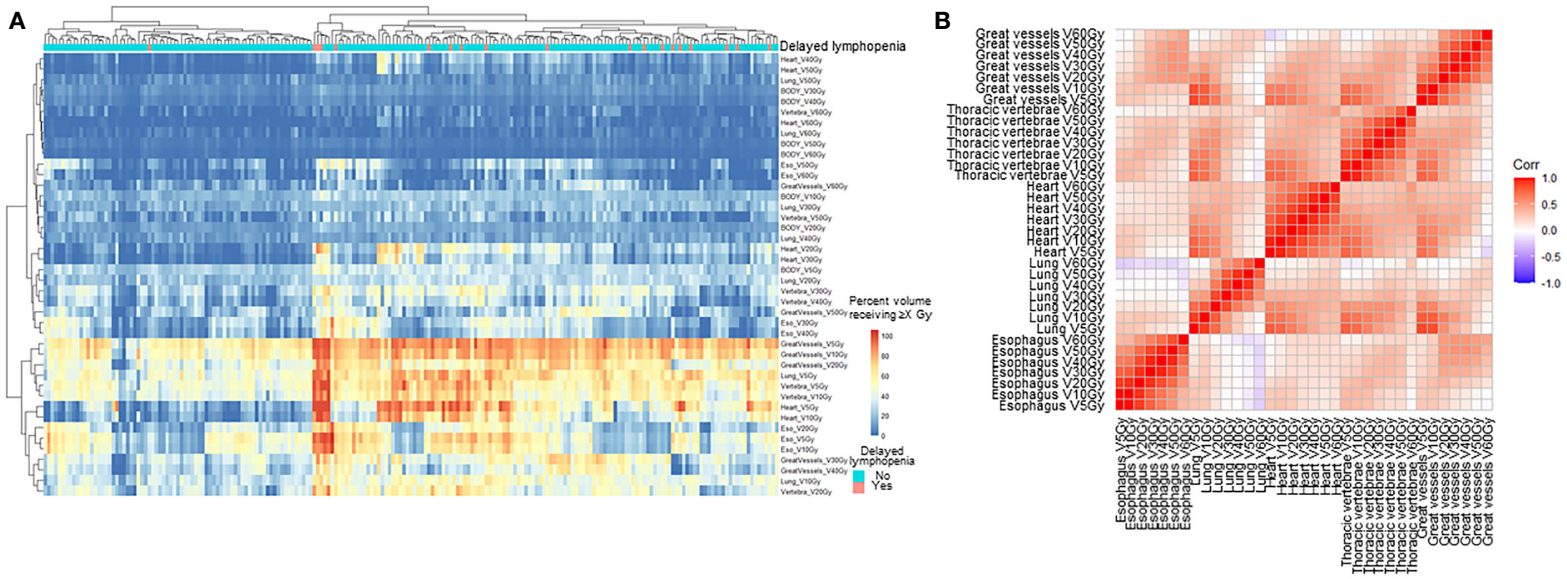
Pattern of dosimetric parameters and delayed lymphopenia. **(A)** Hierarchical clustering analysis of DVH parameters. Heatmap of the percent volume receiving 5-60 Gy for each thoracic organ at risk were clustered by Euclidean method. Column indicates patients, and row represents dosimetric parameters. **(B)** Correlation plot for dosimetric parameters. V5-60Gy, volume receiving 5-60 Gy.

In univariate analysis, older age, longer pack-year smoking history, volumetric modulated arc therapy (VMAT) technique for radiotherapy treatment, and paclitaxel and carboplatin (TC) regimen were associated with delayed lymphopenia (**Table 1**). In terms of blood markers, lower baseline/during-CRT albumin, ALC, and hemoglobin were significantly associated with delayed lymphopenia. To evaluate DVH parameters that can guide clinicians on prevention of delayed lymphopenia, we obtained comprehensive dosimetric parameters including Dmean, Dmax, and V5-V60 for whole body, heart, lung, great vessels, spleen, esophagus, and thoracic vertebral bodies. First, we aimed to demonstrate the distribution of DVH parameters and delayed lymphopenia for the patient cohort. Dosimetric parameters were plotted in heatmaps using the Euclidean clustering method. DVH parameters were divided into two groups. The first comprised low-dose dosimetric parameters (V5, V10, and V20) for great vessels, thoracic vertebrae, heart, lung, and esophagus; the second was the remaining parameters. There was a tendency for more frequent delayed lymphopenia in patients with higher low-dose dosimetric parameters (**Figure 2A**). The cross-correlation plot revealed that low-dose dosimetric parameters for great vessels, thoracic vertebrae, heart, and lung were highly correlated with each other (**Figure 2B**). In univariate analysis, PTV volume; beam on-time; mean heart dose; mean thoracic vertebrae dose; and low-dose dosimetric parameters for esophagus, great vessels, heart, lung, and thoracic vertebrae were associated with delayed lymphopenia (**Table 3**). Because the DVH parameters were all highly correlated with each other, we used ROC analyses to determine the best predictors for delayed lymphopenia (**Supplementary Figure S1**). The area under the ROC curve and the best predictive cut-off values are summarized in **Table 3**. To avoid multicollinearity, the highest AUC variable for each structure was included in the multivariable logistic regression.

**Table 3:** DVH parameters associated with delayed lymphopenia.

Characteristic	Delayed lymphopenia (ALC nadir <500, 4 to 12 weeks after CRT completion)					
	No (N=244)	Yes (N=28)	OR (95% CI)	*P* value ∫	AUC	Cutoff for DL
Lung V5 (%) ∮	58.2 ± 15.4	73.0 ± 13.2	1.074 (1.038-1.117)	0.001	0.76	>67.9%
GreatVessels V5 (%) *	74.6 [63.2;82.4]	83.7 [79.2;88.8]	1.077 (1.030-1.134)	0.003	0.73	>77.2%
Heart V5 (%) *	40.3 [20.5;68.0]	68.2 [48.4;96.2]	1.028 (1.011-1.048)	0.002	0.73	>46.3%
Lung V20 (%) ∮	24.6 ± 7.2	29.8 ± 5.7	1.122 (1.042-1.219)	0.004	0.71	>25.5%
Thoracic vertebrae V10 (%) ∮	50.6 ± 14.7	63.4 ± 17.7	1.053 (1.022-1.087)	0.001	0.71	>53.3%
Heart mean dose (cGy) *	518.4 [20.2;1493.5]	1689.1 [985.4;2400.4]	1.001 (1.000-1.001)	0.002	0.71	>972 cGy
Heart V10 (%) *	31.3 [14.1;51.4]	51.6 [36.5;75.5]	1.028 (1.010-1.047)	0.002	0.71	>38.5%
GreatVessels V10 (%) *	66.7 [56.0;78.2]	77.0 [69.6;84.6]	1.063 (1.022-1.112)	0.004	0.70	>62.5%
Thoracic vertebrae V20 (%) ∮	39.4 ± 15.8	50.8 ± 16.0	1.047 (1.015-1.081)	0.004	0.69	>45.6%
Thoracic vertebrae V5 (%) *	55.1 [47.1;65.9]	61.7 [60.8;73.9]	1.044 (1.014-1.076)	0.004	0.69	>58.8%
Lung V10 (%) ∮	40.0 ± 12.2	49.0 ± 12.1	1.065 (1.022-1.113)	0.004	0.69	>33.1%
Lung V30 (%) ∮	17.4 ± 5.8	20.9 ± 4.9	1.106 (1.021-1.203)	0.015	0.68	>19.1%
Heart V20 (%) *	17.7 [5.8;33.4]	31.8 [18.3;45.5]	1.028 (1.006-1.051)	0.01	0.68	>22.4%
Thoracic vertebrae mean dose (cGy) *	1515.4 [29.1;2077.1]	2156.2 [1154.9;2913.8]	1.001 (1.000-1.001)	0.011	0.67	>1936 cGy
Esophagus V20 (%) *	31.9 [17.7;46.6]	45.8 [29.4;56.1]	1.032 (1.007-1.059)	0.015	0.67	>22.1%
Esophagus V10 (%) *	44.8 [23.2;55.4]	60.0 [36.3;69.2]	1.029 (1.005-1.055)	0.02	0.65	>59.8%
Esophagus V5 (%) *	49.5 [25.9;61.8]	62.6 [39.9;75.8]	1.026 (1.004-1.051)	0.026	0.64	>65.8%
PTV, cc *	337.4 [220.3;513.0]	444.7 [324.7;631.5]	1.002 (1.001-1.004)	0.035	0.64	>365 cc
Total beam on time, sec/course *	2160.0 [1741.8;2453.0]	2295.0 [1982.0;2895.0]	1.000 (1.000-1.001)	0.047	0.58	>3360 sec

OR, odds radio; AUC, area under the curve; DL, delayed lymphopenia; V5-20 Gy, volume receiving 5-20Gy* Expressed as median [interquartile range]; Kruskal-Wallis Rank Sum test were used for comparison ∮ Expressed as mean ± SD; Student t-test were used for comparison ∫ FDR adjusted P value.

**Figure 3:**
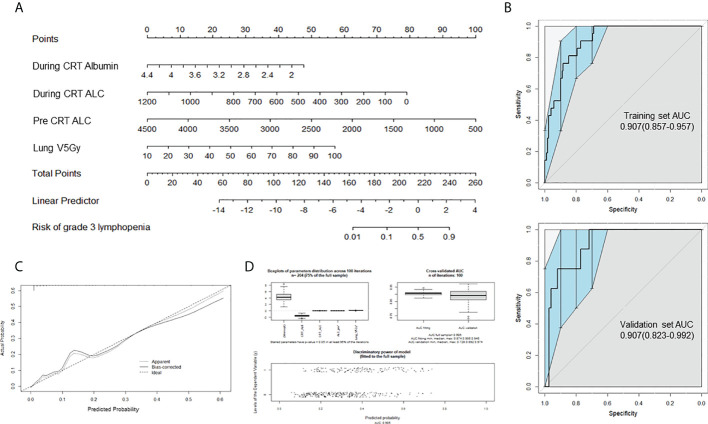
Prediction nomogram for delayed lymphopenia. **(A)** Nomogram for predicting the probability of grade 3 lymphopenia in 4-12 weeks after CRT completion. **(B)** Receiver operating characteristic(ROC) curve AUC of the delayed lymphopenia prediction models, using the training dataset (upper; n = 191) and validation dataset (lower; n = 82) To evaluate the discriminatory ability of the nomogram, we computed the AUC with a 95% CI by using 400 bootstrap resamplings. **(C)** Calibration plot for nomogram. **(D)** Internal validation using 100 iterations of tenfold cross-validation. CRT, chemoradiotherapy; ALC, absolute lymphocyte count; Lung V5 Gy, the percentage of total normal lung volume receiving equal to or greater than 5Gy of radiation; AUC, area under the ROC.

Seventeen variables significantly associated with delayed lymphopenia in univariate analysis (adjusted P value <0.05) were selected for a full multivariable logistic regression analysis. After subsequent stepwise selection, the final model consisted of four independent predictors of delayed lymphopenia, lower baseline and during-CRT ALC, lower during-CRT albumin, and higher lung V5 (**Table 4**). The nomogram developed from the final model is shown in **Figure 3A**. **Figure 3B** presents the results of split-sample model validation. Internal validation of the nomogram was performed using 400 bootstrap resamples, demonstrating a satisfactory calibration curve (**Figure 3C**). The prediction model had good performance with a cross-validated AUC (**Figure 3D**; AUC full sample 0.905; AUC fit median 0.906, range 0.874-0.945; AUC validation median 0.892, range 0.728-0.974).

**Table 4:** Multivariate logistic regression analysis for delayed lymphopenia.

	OR (95% CI)	*P* Value
ALB nadir during CRT, g/dL	0.196 (0.075-0.512)	<0.001
ALC nadir during CRT, g/dL	0.994 (0.990-0.999)	0.010
Baseline ALC, g/dL	0.998 (0.997-0.999)	<0.001
Lung V5, %	1.058 (1.021-1.096)	<0.001

OR, odds radio; ALB, albumin; ALC, absolute lymphocyte count; CRT, chemoradiotherapy; Lung V5, lung volume receiving 5 Gy.

## Discussion

Consolidation immunotherapy becomes a standard treatment after definitive CRT in locally advanced NSCLC. Considering the impact of RIL in the era of immunotherapy, it is important to predict which patients are at high risk for lymphopenia at the time of consolidation immunotherapy. This single-institution, retrospective study assessed a prognostic role and defined predictors for delayed lymphopenia at 4 to 12 weeks after CRT completion when consolidation immunotherapy usually starts. Lung V5, ALC (baseline, nadir during CRT), and albumin nadir during CRT were independent predictors of delayed lymphopenia. We used these predictors to develop a nomogram, which demonstrated an AUC of 0.905.

There is consensus that RIL is associated with poor prognosis (4, 14). Moreover, there is growing evidence that lower ALC can adversely affect immunotherapy response (4, 15–17). During CRT, most patients (up to 90%) experience lymphopenia, but the majority recovers within 1-2 months (9). However, previous studies mostly focused on transient lymphopenia during or early after CRT, and there has been no study that focused on prediction of delayed lymphopenia (9, 14). Friedes et al. reported that post-CRT lymphopenia was associated with more rapid disease progression in patients with NSCLC receiving immunotherapy for cure (18). Other studies on survival impact of post-RT lymphopenia have reported results for small cell lung cancer patients but not for NSCLC patients (16, 19). Here, we report that delayed lymphopenia is associated with detrimental effects on prognosis in locally advanced NSCLC patients receiving definitive CRT. These results emphasize the need for identification of the risk factors and mitigating strategies for delayed lymphopenia in the era of immunotherapy.

For locally advanced NSCLC patients who were treated with definitive CRT, delayed lymphopenia is a quite rare event (10%) and our sample size falls short to make a definite conclusion. Due to the rarity of delayed lymphopenia, imbalanced classification problems also arise for the model prediction. To deal with such limitations, we presented ROC curves with confidence intervals for individual predictors, and the final model performance was evaluated through the bootstrap resampling method. Nevertheless, further studies are needed to validate the prediction model for delayed lymphopenia. However, it is the first study that demonstrates the clinical impact of delayed lymphopenia compared to that of transient lymphopenia. We also made valuable information for prediction of delayed lymphopenia using clustering and correlation analyses with comprehensive DVH profiles. High-dose dosimetric parameters were not associated with lymphopenia, while low-dose DVH (V5-V20) for lung, heart, great vessels, and thoracic vertebrae were predictors for delayed lymphopenia. Furthermore, such factors were highly correlated with each other, and it seems that measuring lung V5 alone could efficiently predict the effect of radiation on lymphopenia without evaluation of the other thoracic anatomical structures.

Numerous studies have investigated the risk factors associated with RIL. Patient-related factors reported to be associated with increased likelihood of RIL include lower baseline ALC (10, 20–24) and older age (11, 23, 25). These factors signify that a small reserve capacity of lymphocytes can be depleted in patients with a poor physical condition (14). In the present study, ALC (baseline/during-CRT) and albumin (during-CRT) nadirs were independent predictors of delayed lymphopenia. Lymphocyte count and albumin are well-known nutritional parameters in hospitalized patients, and malnutrition is a common global cause of lymphopenia (26, 27). In clinical practice, physicians often encounter patients with comorbid RIL and severe malnutrition. However, the association between RIL and nutritional status during CRT has not been fully elucidated. We first showed that albumin level during CRT can predict delayed lymphopenia after CRT. Hypoalbuminemia does not directly indicate malnutrition, as inflammation can also reduce serum albumin level. Further study is needed to determine whether nutritional status during CRT is correlated with RIL and if nutritional support for high-risk patients could ameliorate RIL.

Treatment-related factors such as traditional radiotherapy (vs. SBRT (17) vs. proton therapy (28), longer overall treatment time (24), larger treatment volume (11, 24, 29–31), higher lung V5 (12, 17, 25), heart V5 (11), thoracic vertebrae V20 (11), and mean heart or lung dose (11) have been associated with higher risk of RIL. Emerging evidence suggests that irradiation of circulating lymphocytes in the blood pool plays a significant role in the pathophysiology of RIL (32–35). Although the association was not significant in multivariable analysis, longer overall beam on time and VMAT technique were associated with delayed lymphopenia in univariate analysis. Interestingly, we also found that VMAT led to significantly longer overall beam on-time and higher lung V5 compared to three-dimensional conformal radiation therapy (data not shown). In multivariate analysis, we confirmed that lung V5 was an independent predictor for delayed lymphopenia. Lung V5 was significantly higher in patients who have larger PTV and higher V5-V20 for heart, great vessels, and thoracic vertebral bodies. They tend to be treated with VMAT technique and have severe lymphopenia during CRT. Based on above findings, we can deduce that lung V5 is associated with radiotherapy-related damage to the blood lymphocyte pool, and patients with higher lung V5 seem to have severe RIL and may require a longer time to recover from it.

There are several limitations to our analysis. First, there were the inherent limitations of a retrospective study, including selection bias. Second, the prediction nomogram was validated only internally, and external validation is required. Finally, we analyzed delayed lymphopenia at 4 to 12 weeks after CRT completion when consolidation immunotherapy usually starts. However, there were only 30 (10.9%) patients who were treated with consolidation immunotherapy. Further study is needed to elucidate the prognostic impact of RIL in patients treated with immunotherapy.

Delayed lymphopenia was associated with increased risk of progression and death, while acute lymphopenia was not. We evaluated clinical risk factors for delayed lymphopenia and proposed cut-off values for DVH parameters that predict delayed lymphopenia. Lung V5, baseline ALC, during-CRT ALC, and albumin nadir were independent predictors of delayed lymphopenia. We also developed a nomogram to predict delayed lymphopenia after CRT for patients with locally advanced NSCLC. For risk-reducing interventions before initiation of immunotherapy, our results will help to identify high-risk patients.

## Data availability statement

The original contributions presented in the study are included in the article/**Supplementary Material**. Further inquiries can be directed to the corresponding author.

## Author contributions

Conceptualization, JL, B-HK and XL. Formal analysis, B-HK and XL. Resources, JL, CS, H-CK, HJ, and H-GW. Data curation, B-HK, XL, and JS. Writing-original draft preparation, B-HK. Writing—review and editing, JL. Visualization, B-HK. Supervision, JL. Project administration, JL. All authors have read and agreed to the published version of the manuscript.

## Funding

This work was supported by grants from the Ministry of Science and Information & Communication Technology (NRF-2021R1A2C1095168, NRF-2020M2D9A2092373).

## Conflict of interest

The authors declare that the research was conducted in the absence of any commercial or financial relationships that could be construed as a potential conflict of interest.

## Publisher’s note

All claims expressed in this article are solely those of the authors and do not necessarily represent those of their affiliated organizations, or those of the publisher, the editors and the reviewers. Any product that may be evaluated in this article, or claim that may be made by its manufacturer, is not guaranteed or endorsed by the publisher.
