# Supplementary material for: Prediction and clinical impact of delayed lymphopenia after chemoradiotherapy in locally advanced non-small cell lung cancer
Source: Front Oncol. 2022 Aug 18;12:891221. doi: 10.3389/fonc.2022.891221 (PMC9437922; doi:10.3389/fonc.2022.891221)
Supplement: Supplementary file 1 [file Image_1.pdf]

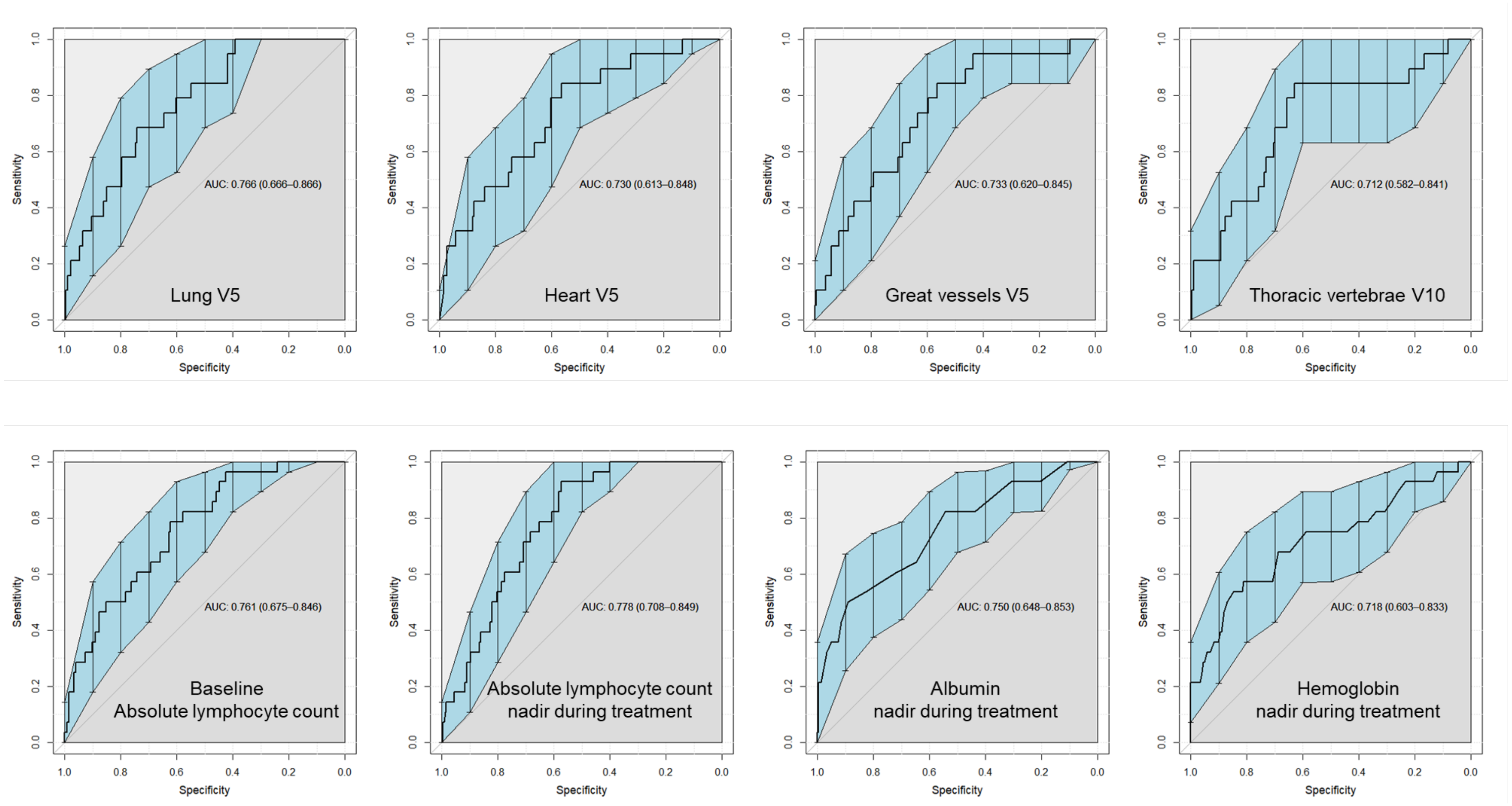

Supplementary Figure 1.

Receiver Operating Characteristic (ROC) and Area Under the Curve (AUC) analyses to determine the best predictors for delayed lymphopenia. We computed the area under the curve with a 95% confidence intervals by using 400 bootstrap resamplings. Abbreviations: V5, the percentage volume receiving equal to or greater than 5Gy of radiation; AUC, area under the receiver operating characteristic curve; V10, the percentage volume receiving equal to or greater than 10Gy of radiation
